# Associations of vegetable and fruit intake, physical activity, and school bullying with depressive symptoms in secondary school students: the mediating role of internet addiction

**DOI:** 10.1186/s12888-024-05867-0

**Published:** 2024-06-04

**Authors:** Lujun Ji, Zhisheng Ren, Jian Chen, Hui Zhao, Xiaofei Zhang, Bai Xue, Dongfeng Zhang

**Affiliations:** 1 Department of Epidemiology and Health Statistics, The School of Public Health of Qingdao University, 308 Ningxia Road, Qingdao, 266071 China; 2 Qingdao Municipal Center for Disease Control and Prevention, Qingdao Institute of Preventive Medicine, Qingdao, China

**Keywords:** Adolescents, Depressive symptoms, Cross-sectional study, Structural equation model

## Abstract

**Background:**

Few studies have simultaneously focused on the associations of vegetable and fruit intake, physical activity, school bullying, and Internet addiction (IA) with depressive symptoms. This study aimed to explore the direct and indirect effects of the above factors on depressive symptoms in adolescents by constructing a structural equation model (SEM).

**Methods:**

This study was conducted in Qingdao from September to November 2021. A total of 6195 secondary school students aged 10–19 years were included in the analysis. Information on all variables was assessed using a self-administered questionnaire. An SEM was constructed with depressive symptoms as the endogenous latent variable, IA as the mediating variable, and vegetable and fruit intake, physical activity, and school bullying as the exogenous latent variables. The standardized path coefficients (*β*) were the direct effects between the latent variables, and the indirect effects were obtained by the product of direct effects between relevant latent variables.

**Results:**

The median value with the interquartile range of depressive symptom scores was 7 (3,12). Vegetable and fruit intake (*β*=-0.100, *P*<0.001) and physical activity (*β*=-0.140, *P*<0.001) were directly negatively related to depressive symptoms. While school bullying (*β*=0.138, *P*<0.001) and IA (*β*=0.452, *P*<0.001) were directly positively related to depressive symptoms. IA had the greatest impact on depressive symptoms. Vegetable and fruit intake, physical activity, and school bullying could not only directly affect depressive symptoms, but also indirectly affect depressive symptoms through the mediating effect of IA, the indirect effects and 95% confidence intervals (CIs) were -0.028 (-0.051, -0.007), -0.114 (-0.148, -0.089) and 0.095 (0.060, 0.157), respectively. The results of the multi-group analysis showed that the SEM we constructed still fit in boy and girl groups.

**Conclusions:**

The results indicated that vegetable and fruit intake, physical activity, school bullying, and IA had a significant direct impact on depressive symptoms, among which IA had the greatest impact. In addition, both vegetable and fruit intake, school bullying, and physical activity indirectly affected depressive symptoms through the mediating effect of IA. The impact of IA on depressive symptoms should be given extra attention by schools and parents. This study provides a scientific and effective basis for the prevention and control of adolescent depressive symptoms.

**Supplementary Information:**

The online version contains supplementary material available at 10.1186/s12888-024-05867-0.

## Background

Depression is a common mental disorder with symptoms such as anhedonia, low mood, fatigue, and insomnia [1], that affects an estimated 280 million individuals worldwide [2]. Adolescence is a crucial period of physical and psychological development, which is prone to various mental health problems, especially depression [3]. The results of a systematic review showed that the prevalence of depressive symptoms among secondary school students in mainland China ranged from 6.2 to 62.8% [4]. Studies have shown that the prevalence of depression in adolescents is increasing year by year, such as the result of a meta-analysis showed that the COVID-19 pandemic has doubled the global prevalence of depression in adolescents [5]. The World Health Organization (WHO) reports that depression is a primary contributor to disease and disability among adolescents [6]. Depression in adolescence is not only a major risk factor for suicidal behavior [7] but also increases the risk of adverse psychological outcomes in adulthood, such as major depression and anxiety [8, 9]. Therefore, it is essential to investigate the modifiable factors of depressive symptoms in adolescents.

Depression is a complex psychological disease, and its pathogenesis is the result of a variety of factors. The relationship between certain lifestyle and behavioral factors such as diet [10], physical activity [11], bullying victimization [12], Internet addiction (IA) [13], and depressive symptoms has attracted extensive attention from researchers. As a common healthy diet, vegetables and fruits are rich in a variety of nutrients with anti-inflammatory and antioxidant properties, such as vitamin C, carotenoids, and folate [14]. Studies have shown that these nutrients have a protective effect against depression [15–17]. Several studies in different regions have shown an inverse association between fruit and vegetable intake and the risk of depressive symptoms [18–20]. Physical activity is defined as any physical movement produced by skeletal muscles that requires energy expenditure [21]. The WHO reports that physical activity is crucial for promoting adolescent physical and mental health [22]. The negative association between physical activity and depressive symptoms in adolescents has been confirmed in several studies [11, 23–25]. . School bullying is a special aggressive behavior, which has the characteristics of intentional, repetitive, and long-term [26], and has gradually become an important public health issue for children and adolescents [27]. A wide range of research has shown there was a positive association between bullying and depressive symptoms [28–30]. IA is defined as excessive or uncontrollable use of the Internet [31], and adolescence is a high-risk period for the emergence of IA [32]. IA may contribute to the development of depressive symptoms by separating individuals from real interpersonal relationships and reducing their social communication [33, 34]. Several studies have shown that the severity of IA was positively correlated with the risk of depressive symptoms [35, 36].

In addition, existing studies have suggested that there may be a certain interaction between the above factors. A longitudinal study found that lower fruit and vegetable consumption predicted a higher risk of IA [37]. Two other randomized controlled trials have shown that interventions based on physical activity may be effective measures to alleviate addictive behaviors, such as IA and smartphone addiction [38, 39]. In addition, a study showed that traumatic experiences may increase the risk of IA [40]. A meta-analysis found that factors such as bullying and interpersonal problems were possible risk factors for online game disorder [41].

Although several studies have explored the relationship between the above factors and depressive symptoms. However, numerous studies focused on one aspect or only considered the impact of individual factors on depressive symptoms. Few studies have simultaneously focused on these factors and further explored the possible interaction between these factors.

**Fig. 1:**
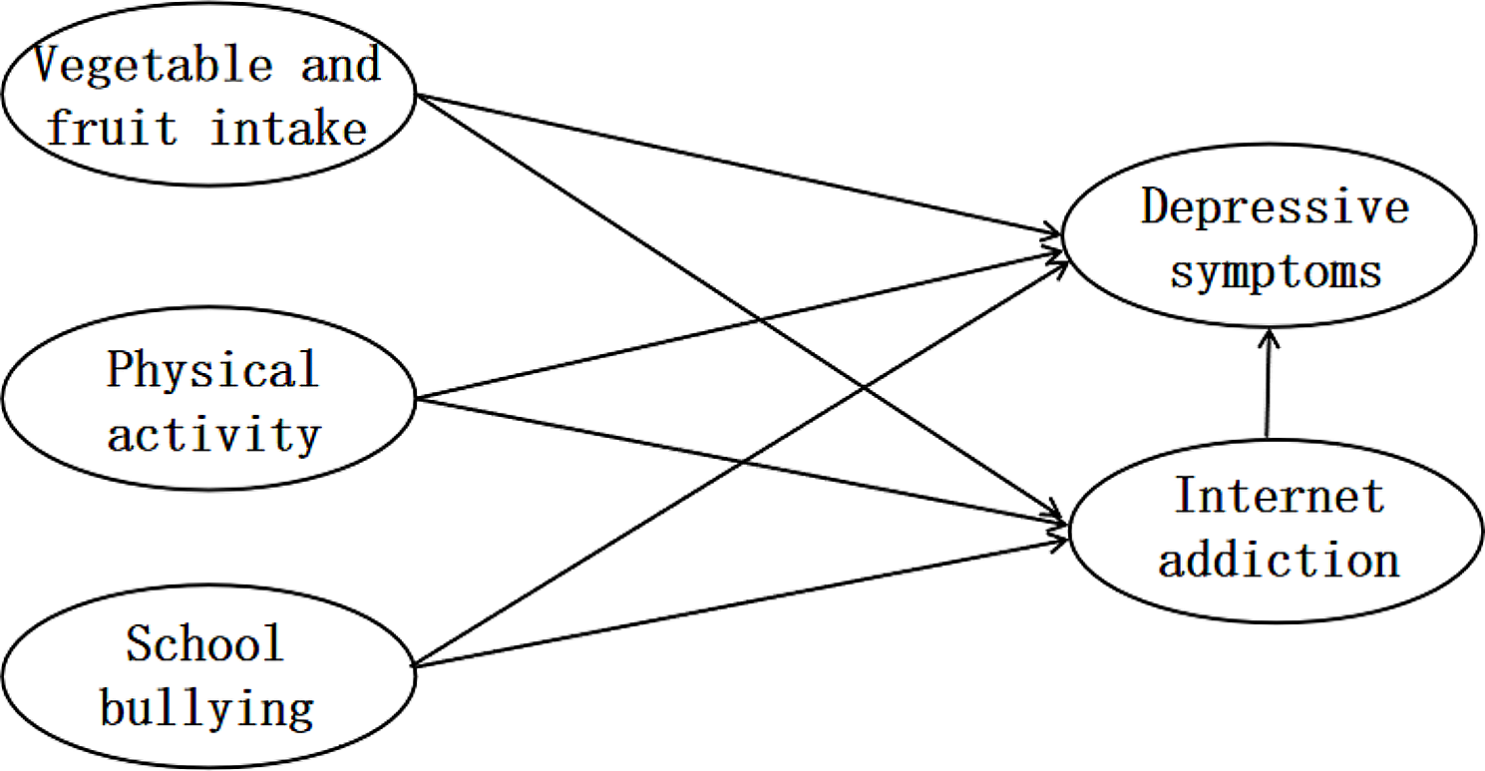
Theoretical model diagram

This study aimed to clarify the direct and indirect effects of the above factors on depressive symptoms in Qingdao secondary school students. A structural equation model (SEM) was constructed with depressive symptoms as the endogenous latent variable, IA as the mediating variable, and vegetable and fruit intake, physical activity, and school bullying as the exogenous latent variables. Based on existing literature, we proposed the following hypotheses. H1: Vegetable and fruit intake is directly negatively correlated with depressive symptoms; H2: Physical activity is directly negatively correlated with depressive symptoms; H3: School bullying is directly positively correlated with depressive symptoms; H4: IA is directly positively correlated with depressive symptoms; H5: IA mediated the link between vegetable and fruit intake and depressive symptoms; H6: IA mediated the link between physical activity and depressive symptoms; H7: IA mediated the link between school bullying and depressive symptoms. The hypothetical theoretical model framework is shown in Fig. 1.

## Methods

### Participants

This cross-sectional study was conducted in Qingdao, Shandong Province from September to November 2021. By using the stratified cluster random sampling method, a total of 12 junior high schools and 12 senior high schools were randomly selected from 10 districts and cities in Qingdao. Each secondary school was sampled by grade, at least 2 or more classes from each grade were randomly selected, and all students in the selected classes participated in the survey. At least 80 students were included in each grade, and at least 240 students were included in each school. In total, 6,395 students completed the questionnaires anonymously. After excluding the students whose questionnaire information was unreliable, a total of 6195 students (3015 girls and 3180 boys) aged 10–19 years were enrolled in our analysis, for an effective questionnaire recovery rate of 96.87%. Among them, 3127 were junior high school students and 3068 were senior high school students. All subjects in this survey signed informed consent forms. This study was approved by the Medical Ethics Committee of the Qingdao Centers for Disease Control and Prevention.

### Measurement

An anonymous self-administered questionnaire survey was conducted on the participants, and the questionnaire was uniformly distributed by the investigators. The questionnaire was used to evaluate the information on vegetable and fruit intake, physical activity, school bullying, IA, and depressive symptoms.

#### Depressive symptoms

The Center for Epidemiological Studies Depression (CES-D) Scale was used to assess depressive symptoms [42]. The scale consisted of 20 items, and the scores ranged from 0 to 60. More severe depressive symptoms were indicated by higher scores. In addition, the CES-D scale contains four factors, namely, positive affect, depressive affect, interpersonal relationships, and somatic symptoms. Based on the findings of a previous study [43], we combined positive affect and depressive affect in the CES-D scale into one factor, termed affect symptoms. Then, we used the item parceling method to package the items for the three factors of affect symptoms, somatic symptoms, and interpersonal relationships [44, 45]. In this study, the Cronbach’s α of the CES-D scale was 0.868. The KMO value of the scale was 0.942, and the result of Bartlett’s spherical test was significant (*P*<0.001).

#### Vegetable and fruit intake

The frequency and variety of fresh vegetable or fruit intake were investigated using the following questions. Participants were asked how many times they had eaten fresh vegetables or fruit in the last 7 days. The options available for participants to answer were “never eat”, “less than once a day”, “once a day”, or “twice a day or more”. In addition, participants were asked how many kinds of fresh vegetables or fruits they usually eat each day. The options available for participants to answer were “never eat”, “less than one kind a day”, “one kind a day”, or “two kinds a day”.

#### Physical activity

The following 2 questions were used to assess physical activity. Participants were asked how many days in the past 7 days/weekends or holidays they had at least 60 mins of moderate-to-high intensity exercise. Moderate-to-high intensity exercise was defined as exercise that accelerates participants’ breathing or heartbeat, such as running, basketball, swimming, aerobics, and lifting heavy objects. The options available for participants to answer were “hardly do”, “a few days”, “half of the days”, “most days”, or “all of the days”.

#### School bullying

The scale used to assess school bullying in our study was adapted from the Olweus Bully/Victim Questionnaire [46], and the Chinese version has been validated and widely used among Chinese adolescents [47, 48]. The scale consists of 6 questions. Each question tested whether participants had experienced some form of bullying in or around school in the past 30 days. The detailed information is presented in Table 1. The options available for participants to answer were “never”, “sometimes”, or “often”. The Cronbach’s α of the scale in our study was 0.756. The KMO value of the scale was 0.822, and the result of Bartlett’s spherical test was significant (*P*<0.001).

**Table 1:** Latent variables and observed variables

Latent variables (Abbreviation)	Observed variables (Abbreviation)
Vegetable and fruit intake (VF)	How many times have you eaten fresh fruit in the last 7 days? (VF1)
	How many kinds of fresh fruit do you usually eat each day? (VF2)
	How many times have you eaten fresh vegetables in the last 7 days? (VF3)
	How many kinds of fresh vegetables do you usually eat each day? (VF4)
Physical activity (PA)	In the past 7 days, how many days have you had at least 60 min of moderate to high-intensity exercise? (PA1)
	On weekends or holidays, how many days have you had at least 60 min of moderate to high-intensity exercise? (PA2)
Internet addiction (IA)	Often on the Internet, even if not on the Internet, the mind has always emerged with the Internet-related things. (IA1)
	Once the Internet is not available, may feel uncomfortable or unwilling to do other things, and the Internet relieves it. (IA2)
	Increase online time to get satisfaction. (IA3)
	Loss of interest in other recreational activities (hobbies, meeting friends) because of the Internet use. (IA4)
	Many times want to stop the Internet, but always can’t control. (IA5)
	Unable to finish homework or play truant from school because of the Internet. (IA6)
	Hide the facts of the Internet from parents, teachers and classmates. (IA7)
	Knowing the negative consequences (lack of sleep, being late for class, and arguing with parents) and continuing to use the Internet. (IA8)
	In order to escape from reality, get rid of difficulties, depression, helplessness and anxiety to surf the Internet. (IA9)
School bullying (SB)	Being maliciously teased. (SB1)
	Being asked for the property. (SB2)
	Being intentionally excluded from or isolated from group activities. (SB3)
	Being threatened and intimidated. (SB4)
	Being beaten, kicked, pushed, squeezed, or locked in the house. (SB5)
	Being made fun of for physical defects or looks. (SB6)
Depressive symptoms (DP)	Affect Symptoms (AS)
	Somatic Symptoms (SS)
	Interpersonal Relationships (IR)

#### Internet addiction

The scale used to assess IA in our study was adapted from Young’s Diagnostic Questionnaire (YDQ) [31]. The Chinese version of the YDQ scale has good reliability and validity in the adolescent population [38, 49, 50]. The scale consists of 9 questions. Each question tested whether participants had experienced a certain symptom of IA while surfing the Internet (including using mobile phones, tablets, computers, or other electronic devices). The detailed information is presented in Table 1. The options available for participants to answer were “No” or “Yes”. The Cronbach’s α of the scale in our study was 0.789. The KMO value of the scale was 0.884, and the result of Bartlett’s spherical test was significant (*P*<0.001).

### Statistical analysis

The Harman’s single-factor test was used to examine the common method bias of the data. Confirmatory factor analysis (CFA) was performed to determine the observed variables under each latent variable and to assess the structural, convergent, and discriminant validity of the measurement model. The specific definitions of the observed variables under each latent variable and the corresponding abbreviations are presented in Table 1. SEM was constructed to test hypotheses H1-H7, and the Asymptotic distribution-free (ADF) method was used to estimate the model because the data deviated significantly from the normal distribution (Additional file 1: Table S1) [51]. Based on the modification indices, the SEM was modified by adding the covariance between the error terms. The bootstrap method (repeated sampling 5000 times) was used to test the mediating effect of IA, and a bias-corrected 95% confidence interval was calculated to test the significance of the mediating effect. In the SEM, the direct effect was the standardized path coefficient (*β*) between the latent variables, and the indirect effect was obtained by the product of *β* between the corresponding latent variables. Finally, we further conducted a gender-based multi-group analysis.

The chi-square/degrees of freedom (CMIN/DF), goodness-of-fit index (GFI), adjusted goodness-of-fit index (AGFI), comparative fit index (CFI), incremental fit index (IFI), Tucker-Lewis index (TLI), and root mean square error of approximation (RMSEA) were used to assess the fitting of the SEM [52].

All statistical analyses were performed using SPSS 24.0 and AMOS 24.0. *P* < 0.05 was considered to indicate statistical significance.

## Results

### Common method bias test

The result of Harman’s single-factor test showed that the variation interpretation rate of the first factor with an eigenvalue greater than 1 was 21.82%, which was lower than the critical standard of 40%. It was considered that there was no serious common method bias in the data of our study.

### Characteristics of students

Among the 6195 secondary school students, the mean age was 15.1 years (SD: 1.8), ranging from 10.1 to 19.3 years. Of these students, 3015 (48.7%) were girls, 3180 (51.3%) were boys; 3127 (50.5%) were junior high school students, and 3068 (49.5%) were senior high school students. The median and interquartile range of students’ depressive symptom scores was 7 (3,12).

### Confirmatory factor analysis

In the CFA, we removed observed variables with factor loadings less than 0.5 (including SB4-SB6 and IA7-IA9) (Additional file 2: Figure S1). Although a few values did not reach the ideal standard, the overall results showed that the measurement model had acceptable structural, convergent, and discriminant validity (Additional file 1: Tables S2-S4).

### Structural equation model results

**Fig. 2:**
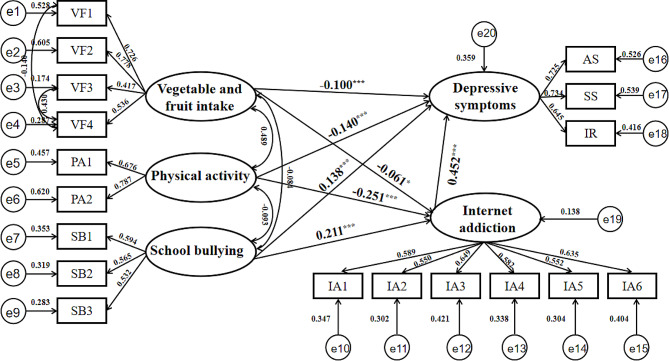
Final structural equation model diagram. VF vegetable and fruit intake, PA physical activity, SB school bullying, IA Internet addiction, AS affect symptoms, SS somatic symptoms, IR interpersonal relationships, e error item. ^*^p < 0.05, ^***^p < 0.001

The final SEM image is shown in Fig. 2. The results of the goodness-of-fit test indicated that the final SEM fit well. The fit indices of the initial and final structural equation models and the reference values are shown in Table 2.

**Table 2:** Model fit indices for the initial and final structural equation models

Fit index	Reference value	Initial model	Final model
CMIN/DF	<5	9.686	4.678
RMSEA	<0.05	0.037	0.024
GFI	>0.9	0.946	0.974
AGFI	>0.9	0.926	0.964
CFI	>0.9	0.764	0.902
IFI	>0.9	0.766	0.902
TLI	>0.9	0.712	0.878

CMIN chi-square value, DF degrees of freedom, RMSEA root mean square error of approximation, GFI goodness-of-fit index, AGFI adjusted goodness-of-fit index, NFI normed fit index, CFI comparative fit index, IFI incremental fit index, TLI Tucker-Lewis index

SEM results showed that vegetable and fruit intake (*β*=-0.100, *P*<0.001) and physical activity (*β*=-0.140, *P*<0.001) were directly negatively related to depressive symptoms. School bullying (*β* = 0.138, *P*<0.001) and IA (*β* = 0.452, *P*<0.001) were directly positively related to depressive symptoms. IA had the greatest impact on depressive symptoms. The results are shown in Table 3.

**Table 3:** Direct effects for the final structural equation model

Path	*β*	SE	CR
H1: Vegetable and fruit intake → Depressive symptoms	-0.100^***^	0.017	-4.585
H2: Physical activity → Depressive symptoms	-0.140^***^	0.008	-6.106
H3: School bullying → Depressive symptoms	0.138^***^	0.170	3.342
H4: Internet addiction → Depressive symptoms	0.452^***^	0.065	15.460

*β* Standardized path coefficients, SE standard error, CR critical ratio, H hypothesis. ^***^p < 0.001

The results of the mediation analysis are presented in Table 4. IA mediated a significant association between fruit and vegetable intake and depressive symptoms, and the indirect effect was -0.028 (95%CI: -0.051, -0.007). The association between physical activity and depressive symptoms was also mediated by IA, and the indirect effect was -0.114 (95%CI: -0.148, -0.089). In addition, IA mediated the association between school bullying and depressive symptoms, the indirect effect was 0.095 (95%CI: 0.060, 0.157).

**Table 4:** Mediating effect of internet addiction in the association between vegetable and fruit intake, physical activity, school bullying and depressive symptoms

Path	Effect	*β*	Bias-corrected 95%CI	
			Lower	Upper
H5: Vegetable and fruit intake → Internet addiction → Depressive symptoms	Indirect	-0.028^**^	-0.051	-0.007
	Direct	-0.100^**^	-0.146	-0.051
	Total	-0.128^***^	-0.179	-0.078
H6: Physical activity → Internet addiction → Depressive symptoms	Indirect	-0.114^***^	-0.148	-0.089
	Direct	-0.140^**^	-0.186	-0.092
	Total	-0.254^***^	-0.302	-0.207
H7: School bullying → Internet addiction → Depressive symptoms	Indirect	0.095^***^	0.060	0.157
	Direct	0.138^**^	0.039	0.233
	Total	0.233^***^	0.146	0.337

*β* Standardized path coefficient, CI confidence interval, H hypothesis. ^**^p < 0.01, ^***^p < 0.001

In this study, the AIC value and ECVI value of the structural weights model are the smallest (Additional file 1: Table S5), which is the optimal model. The results of the structural weights model in the multi-group analysis showed that the SEM we constructed still applies to the boy and girl groups (Additional file 2: Figs. S2 and S3).

## Discussion

In this study of 6195 secondary school students in Qingdao, we found that vegetable and fruit intake and physical activity were directly negatively correlated with depressive symptoms, while school bullying and IA were directly positively correlated with depressive symptoms. Among them, IA had the greatest impact on depressive symptoms. In addition, our results suggested that vegetable and fruit intake, physical activity, and school bullying could not only directly affect depressive symptoms, but also indirectly affect depressive symptoms through the mediating effect of IA.

Our results indicated that vegetable and fruit intake was correlated with a decreased risk of depressive symptoms, which was consistent with prior research findings [19, 20, 53, 54]. Vegetables and fruits are rich in a variety of vitamins, minerals, and phytochemicals, such as vitamin C, potassium, anthocyanins, and carotenoids [55], which have numerous beneficial biological effects on human health. Studies have shown that nutrients such as vitamin C, carotenoids, selenium, and phenols play a protective role against depressive symptoms by reducing oxidative stress levels through their antioxidant effects [15, 56, 57]. Furthermore, numerous studies have shown that B-vitamin deficiencies, such as folate and vitamin B6, may lead to an increase in homocysteine levels, which in turn increased the risk of depression [58, 59].

Our study also revealed that physical activity was negatively related to the risk of depressive symptoms. Consistent with our findings, the negative relationship between physical activity and depressive symptoms has been widely demonstrated in previous research [60]. A review discovered that physical activity may exert its antidepressant effect through biological mechanisms such as stimulating neural plasticity, reducing inflammation and oxidative stress levels, and social psychological mechanisms, such as improving self-esteem and physical self-perception, enhancing social support, improving self-efficacy [61].

In addition, we found a positive correlation between school bullying and the odds of depressive symptoms. The link between school bullying and mental health issues has received extensive attention from researchers. Consistent with our findings, a cross-sectional study of 2,155 students found that participants who were bullied had higher levels of depression [28]. Another meta-analysis suggested that children and adolescents who experience bullying were associated with a higher incidence of depression, with an odd ratio of 2.77 [62]. A study used an SEM to explore the relationship between bullying and depressive symptoms in 4,289 Italian adolescents and found a significant positive association [63]. School bullying may have a strong negative impact on victims, such as reduced self-esteem, emotional disorders, loneliness, and a series of stress response changes, which in turn lead to depressive symptoms [12, 64–66].

Our results suggested that IA was positively associated with the risk of depressive symptoms. Findings from a meta-analysis suggested that adolescents with IA had a greater risk of depression [67]. A study analyzed the survey data of 11,813 adolescents in Shandong Province and found that long-term use of mobile phones was positively correlated with the risk of depressive symptoms [68]. In addition, a cross-sectional study based on SEM found that depressive symptoms mediated the association between IA and non-suicidal self-injury [69], which also supported our findings to some extent.

Our results not only showed the direct effects of vegetable and fruit intake, physical activity, and school bullying on depressive symptoms but also found that the above factors played their indirect effects through the mediating role of IA. Several studies have demonstrated that inadequate intake of fruits and vegetables was positively associated with the risk of IA [37, 70]. Adequate intake of fruits and vegetables may alleviate depressive symptoms by reducing the risk of IA. Several studies have indicated that physical activity may be an effective way to reduce IA [71, 72]. Physical activity may alleviate the degree of IA through mechanisms such as regulating the central and autonomic nervous system, enhancing self-efficacy and self-control, and improving coping styles [73–75]. Therefore, physical activity may reduce the risk of depressive symptoms by reducing the degree of IA. Victims of bullying tend to feel inferior, helpless, and at higher risk of interpersonal barriers [76]. This may lead them to seek comfort and emotional support through virtual worlds such as the Internet to escape the pressures of real life, which in turn causes social isolation and increases the risk of depressive symptoms. A cross-sectional study from rural Henan Province, China showed that being bullied was related to a greater risk of IA [77]. Consistent with our findings, a study of 2,022 junior high school students showed that the association between bullying victimization and depressive symptoms was partially mediated by IA [48].

Among the above-influencing factors, IA has the largest effect on depressive symptoms, and IA also played a mediating role in the association between vegetable and fruit intake, physical activity, school bullying, and depressive symptoms. With the significant increase in the prevalence of IA among adolescents, the impact of IA on depressive symptoms deserves additional attention. Preventing students’ IA requires the efforts of schools, families, and society. Society and schools suggest preventing students’ IA by controlling the enrollment rate of mobile phones, carrying out various recreational and sports activities, and enriching outdoor activities, so as to reduce the adverse effects of IA on mental health.

Our study has the following strengths. First, the participants in this study were selected from ten districts and cities in Qingdao, which had a large sample and good representativeness. Second, thorough quality control was implemented during the investigation. Third, the acceptability and feasibility of the questionnaire were good, which ensured a high response rate. However, this study has the following limitations. First, this study was cross-sectional, and no causal relationships could be inferred. Second, all the variables in this study were obtained through self-administered questionnaires, which may cause information bias. Third, the assessment of vegetable and fruit intake and physical activity is not based on the maturity scale, which may affect the validity of the results to some extent. Fourth, depressive symptoms may also be affected by sleep status, smoking, and other factors. The influencing factors included in our study are not comprehensive. In the future, it is still necessary to further explore the influencing factors of depressive symptoms and the possible interaction between factors as comprehensively as possible.

## Conclusions

Our results indicated that vegetable and fruit intake and physical activity were directly negatively related to depressive symptoms, while school bullying and IA were directly positively related to depressive symptoms. In addition, both vegetable and fruit intake, school bullying, and physical activity indirectly affected depressive symptoms through the mediating effect of IA. The findings on the mediating role of IA extend those of previous studies and reveal potential mechanisms and interactive relationships among vegetable and fruit intake, physical activity, school bullying, IA, and depressive symptoms. The greatest impact of IA on depressive symptoms suggests that we should pay extra attention and take effective measures to prevent adolescent IA to reduce the risk of depressive symptoms. In a word, schools and parents should keep a watchful eye on the mental health of adolescent students from various aspects as much as possible. This study has important implications for developing effective strategies to reduce the risk of depressive symptoms in adolescents.

### Electronic supplementary material

Below is the link to the electronic supplementary material.

Supplementary Material 1

Supplementary Material 2

## Data Availability

The datasets generated and/or analyzed during the current study are not publicly available due to ethic issues involving participant’s privacy but are available from the corresponding author on reasonable request.

## Abbreviations

CIs: confidence interval
IA: Internet addiction
WHO: World Health Organization
SEM: structural equation model
CES-D: Center for Epidemiological Studies Depression Scale
CFA: Confirmatory Factor Analysis
CMIN/DF: Chi-square/degrees of freedom
GFI: goodness-of-fit index
AGFI: adjusted goodness-of-fit index
CFI: comparative fit index
IFI: incremental fit index
TLI: Tucker-Lewis index
RMSEA: root mean square error of approximation
ADF: Asymptotic distribution-free
