# Supplementary material for: Associations of vegetable and fruit intake, physical activity, and school bullying with depressive symptoms in secondary school students: the mediating role of internet addiction
Source: BMC Psychiatry. 2024 Jun 4;24:419. doi: 10.1186/s12888-024-05867-0 (PMC11151523; doi:10.1186/s12888-024-05867-0)
Supplement: Supplementary file 1 — Supplementary Material 1 [file 12888_2024_5867_MOESM1_ESM.docx]

**Table S1.** Normality test of observed variables.

| **Variable** | **min** | **max** | **skew** | **c.r.** | **kurtosis** | **c.r.** |  |
| --- | --- | --- | --- | --- | --- | --- | --- |
| VF1 | 1 | 4 | -0.588 | -18.881 | -0.206 | -3.313 |  |
| VF2 | 1 | 4 | -0.115 | -3.698 | -0.702 | -11.273 |  |
| VF3 | 1 | 4 | -1.581 | -50.788 | 1.935 | 31.092 |  |
| VF4 | 1 | 4 | -0.893 | -28.7 | 0.082 | 1.316 |  |
| PA1 | 1 | 5 | -0.046 | -1.489 | -1.106 | -17.774 |  |
| PA2 | 1 | 5 | -0.023 | -0.734 | -1.261 | -20.255 |  |
| SB1 | 1 | 3 | 4.193 | 134.746 | 18.464 | 296.651 |  |
| SB2 | 1 | 3 | 6.502 | 208.911 | 45.488 | 730.819 |  |
| SB3 | 1 | 3 | 6.662 | 214.065 | 48.386 | 777.378 |  |
| IA1 | 0 | 1 | 2.497 | 80.226 | 4.234 | 68.018 |  |
| IA2 | 0 | 1 | 3.936 | 126.485 | 13.495 | 216.812 |  |
| IA3 | 0 | 1 | 3.225 | 103.643 | 8.404 | 135.017 |  |
| IA4 | 0 | 1 | 2.956 | 94.988 | 6.739 | 108.265 |  |
| IA5 | 0 | 1 | 4.265 | 137.056 | 16.193 | 260.161 |  |
| IA6 | 0 | 1 | 3.051 | 98.034 | 7.308 | 117.413 |  |
| AS | 0 | 3 | 1.304 | 41.895 | 1.978 | 31.786 |  |
| SS | | 0 | 3 | 1.908 | 61.302 | 4.443 | 71.375 |
| IR | 0 | 3 | 3.334 | 107.132 | 11.938 | 191.803 |  |

c.r. critical ratio, VF vegetable and fruit intake, PA physical activity, SB school bullying, IA Internet addiction, AS affect symptoms, SS somatic symptoms, IR interpersonal relationships

**Table S2.** Results of structural validity analysis.

| **Fit index** | **Reference value** | **Measurement model** |
| --- | --- | --- |
| CMIN/DF | ＜5 | 9.686 |
| RMSEA | ＜0.05 | 0.037 |
| GFI | ＞0.9 | 0.946 |
| AGFI | ＞0.9 | 0.926 |
| CFI | ＞0.9 | 0.764 |
| IFI | ＞0.9 | 0.766 |
| TLI | ＞0.9 | 0.712 |

CMIN chi-square value, DF degrees of freedom, RMSEA root mean square error of approximation, AGFI adjusted goodness-of-fit index, GFI goodness-of-fit index, CFI comparative fit index, IFI incremental fit index, TLI Tucker-Lewis index

**Table S3.** Results of convergent validity analysis.

| **Path** | | | **Unstandardized coefficients** | **SMC** | **Standardized weights (Factor loading)** | **CR** | **AVE** |
| --- | --- | --- | --- | --- | --- | --- | --- |
| VF1 | <--- | VF | 1.000 | 0.440 | 0.663 | 0.747 | 0.429 |
| VF2 | <--- | VF | 1.320 | 0.609 | 0.781 |  |  |
| VF3 | <--- | VF | 0.605 | 0.272 | 0.522 |  |  |
| VF4 | <--- | VF | 0.931 | 0.394 | 0.628 |  |  |
| PA1 | <--- | PA | 1.000 | 0.469 | 0.685 | 0.701 | 0.540 |
| PA2 | <--- | PA | 1.192 | 0.611 | 0.782 |  |  |
| SB1 | <--- | SB | 1.000 | 0.330 | 0.574 | 0.591 | 0.325 |
| SB2 | <--- | SB | 0.684 | 0.327 | 0.572 |  |  |
| SB3 | <--- | SB | 0.714 | 0.318 | 0.564 |  |  |
| IA1 | <--- | IA | 1.000 | 0.359 | 0.599 | 0.774 | 0.364 |
| IA2 | <--- | IA | 0.675 | 0.337 | 0.581 |  |  |
| IA3 | <--- | IA | 0.878 | 0.424 | 0.651 |  |  |
| IA4 | <--- | IA | 0.845 | 0.337 | 0.580 |  |  |
| IA5 | <--- | IA | 0.597 | 0.311 | 0.558 |  |  |
| IA6 | <--- | IA | 0.885 | 0.414 | 0.644 |  |  |
| AS | <--- | DP | 1.000 | 0.516 | 0.718 | 0.734 | 0.486 |
| SS | <--- | DP | 0.959 | 0.536 | 0.732 |  |  |
| IR | <--- | DP | 0.706 | 0.408 | 0.638 |  |  |

SMC squared multiple correlations, CR composite reliability, AVE average variance extracted, VF vegetable and fruit intake, PA physical activity, SB school bullying, IA Internet addiction, AS affect symptoms, SS somatic symptoms, IR interpersonal relationships, DP depressive symptoms

**Table S4.** Results of discriminant validity analysis.

|  | **DP** | **IA** | **SB** | **PA** | **VF** |
| --- | --- | --- | --- | --- | --- |
| **DP** | 1 |  |  |  |  |
| **IA** | 0.560^***^ | 1 |  |  |  |
| **SB** | 0.293^***^ | 0.235^***^ | 1 |  |  |
| **PA** | -0.352^***^ | -0.303^***^ | -0.109^***^ | 1 |  |
| **VF** | -0.277^***^ | -0.207^***^ | -0.088^***^ | 0.511^***^ | 1 |
| **AVE** | 0.486 | 0.364 | 0.325 | 0.540 | 0.429 |
| **Square root of AVE** | 0.697 | 0.603 | 0.570 | 0.735 | 0.655 |

DP depressive symptoms, IA Internet addiction, SB school bullying, PA physical activity, VF vegetable and fruit intake, AVE average variance extracted, ^***^ *P*＜0.001

**Table S5.** The AIC and ECVI values of each model in multi-group analysis.

| **Model** | **AIC** | **ECVI** |
| --- | --- | --- |
| Unconstrained | 969.878 | 0.157 |
| Measurement weights | 965.719 | 0.156 |
| Structural weights | 956.319 | 0.154 |
| Structural covariances | 977.305 | 0.158 |
| Structural residuals | 1005.11 | 0.162 |
| Measurement residuals | 1051.577 | 0.170 |
