# Supplementary material for: Associations of vegetable and fruit intake, physical activity, and school bullying with depressive symptoms in secondary school students: the mediating role of internet addiction
Source: BMC Psychiatry. 2024 Jun 4;24:419. doi: 10.1186/s12888-024-05867-0 (PMC11151523; doi:10.1186/s12888-024-05867-0)
Supplement: Supplementary file 2 — Supplementary Material 2 [file 12888_2024_5867_MOESM2_ESM.docx]

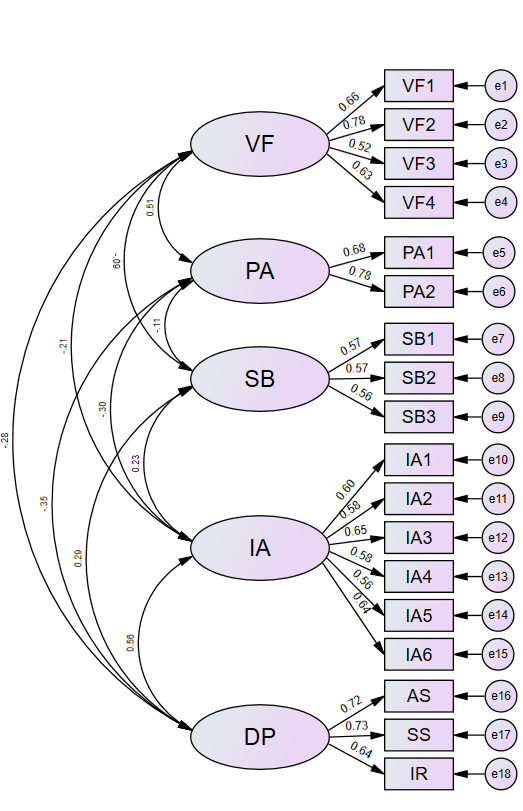


**Figure S1.** Confirmatory factor analysis model diagram. VF: vegetable and fruit intake, PA: physical activity, SB: school bullying, IA: Internet addiction, DP: depressive symptoms, AS: affect symptoms, SS: somatic symptoms, IR: interpersonal relationships, e: error term.


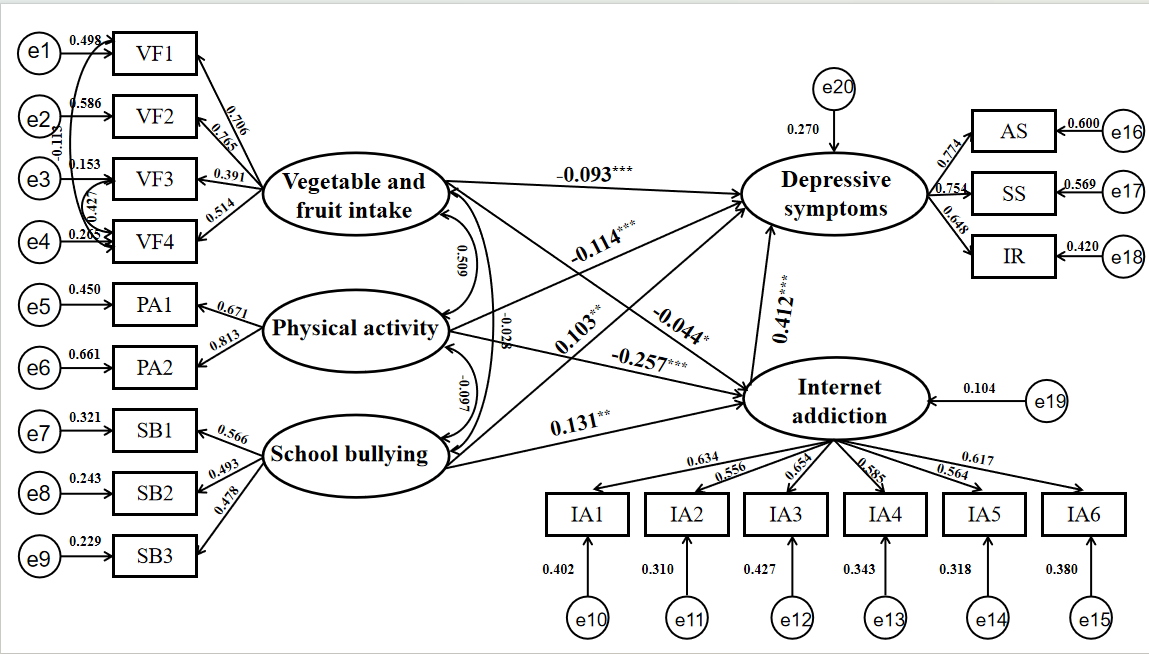


**Figure S2.** Final structural equation model diagram in girls. VF vegetable and fruit intake, PA physical activity, SB school bullying, IA Internet addiction, AS affect symptoms, SS somatic symptoms, IR interpersonal relationships, e error item. ^*^ *p* < 0.05, ^**^ *p* < 0.01, ^***^ *p* < 0.001.


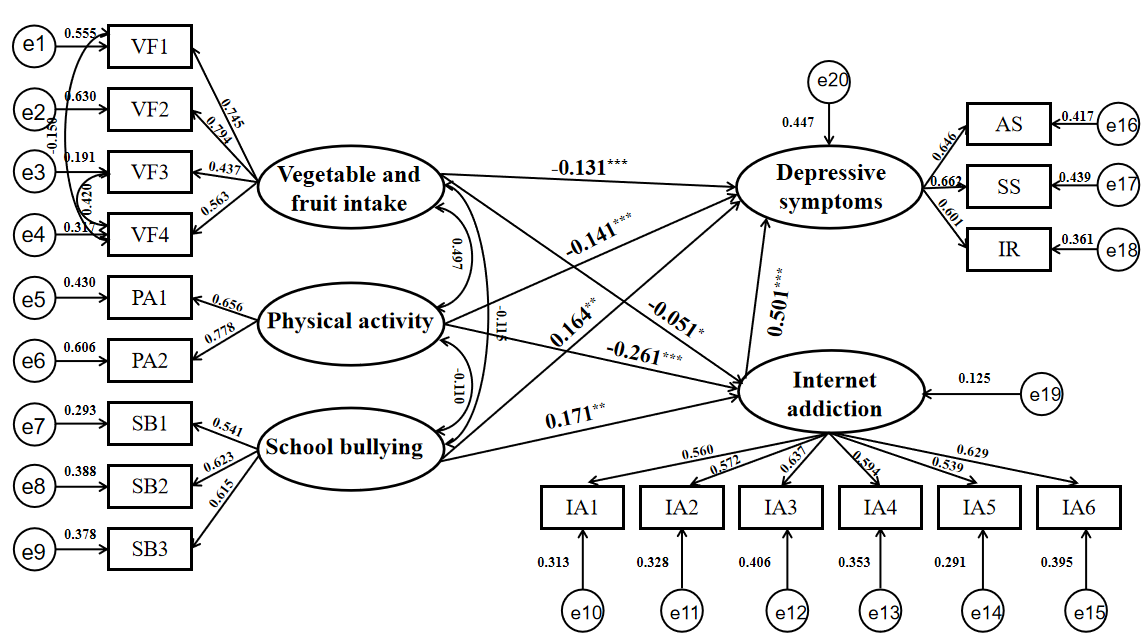


**Figure S3.** Final structural equation model diagram in boys. VF vegetable and fruit intake, PA physical activity, SB school bullying, IA Internet addiction, AS affect symptoms, SS somatic symptoms, IR interpersonal relationships, e error item. ^*^ *p* < 0.05, ^**^ *p* < 0.01, ^***^ *p* < 0.001.
